# Gut microbiome profiling of neonates using Nanopore MinION and Illumina MiSeq sequencing

**DOI:** 10.3389/fmicb.2023.1148466

**Published:** 2023-05-15

**Authors:** Teahyen Cha, Hoo Hugo Kim, Jihyun Keum, Min-Jin Kwak, Jae Yong Park, Jeong Kyu Hoh, Chang-Ryul Kim, Byong-Hun Jeon, Hyun-Kyung Park

**Affiliations:** ^1^Department of Pediatrics, Hanyang University College of Medicine, Seoul, Republic of Korea; ^2^Department of Earth Resources and Environmental Engineering, Hanyang University, Seoul, Republic of Korea; ^3^Department of Obstetrics and Gynecology, Hanyang University College of Medicine, Seoul, Republic of Korea; ^4^Department of Agricultural Biotechnology and Research Institute of Agriculture and Life Sciences, Seoul National University, Seoul, Republic of Korea; ^5^Division of Microbiome, Int-Gen Company, Seoul, Republic of Korea

**Keywords:** gut microbiome, neonates, Illumina MiSeq^®^, Nanopore MinION, preterm infants

## Abstract

This study aimed to evaluate the difference in gut microbiomes between preterm and term infants using third-generation long-read sequencing (Oxford Nanopore Technologies, ONT) compared with an established gold standard, Illumina (second-generation short-read sequencing). A total of 69 fecal samples from 51 term (T) and preterm (P) infants were collected at 7 and 28 days of life. Gut colonization profiling was performed by 16S rRNA gene sequencing using ONT. We used Illumina to validate and compare the patterns in 13 neonates. Using bioinformatic analysis, we identified features that differed between P and T. Both T1 and P1 microbiomes were dominated by *Firmicutes* (*Staphylococcus* and *Enterococcus*), whereas sequentially showed dominant transitions to *Lactobacillus* (*p* < 0.001) and *Streptococcus* in T2 (*p* = 0.001), and pathogenic bacteria (*Klebsiella*) in P2 (*p* = 0.001). The abundance of beneficial bacteria (*Bifidobacterium* and *Lactobacillus*) increased in T2 (*p* = 0.026 and *p* < 0.001, respectively). These assignments were correlated with the abundance at the species-level. Bacterial α-diversity increased in T (*p* = 0.005) but not in P (*p* = 0.156), and P2 showed distinct β-diversity clustering than T2 (*p* = 0.001). The ONT reliably identified pathogenic bacteria at the genus level, and taxonomic profiles were comparable to those identified by Illumina at the genus level. This study shows that ONT and Illumina are highly correlated. P and T had different microbiome profiles, and the α- and β-diversity varied. ONT sequencing has potential for pathogen detection in neonates in clinical settings.

## Introduction

Perturbations in the infant gut microbiome during early life affect growth, development, and long-term health (Sherman, 2010; Carl et al., 2014; Tarr and Warner, 2016). Preterm infants have a physiologically and anatomically immature gastrointestinal tract that is known to be more permeable than that of term infants, and their microbiota colonization is challenged by environmental factors, such as antibiotic use, hospital stay, and enteral feeding (Dominguez-Bello et al., 2010; Fallani et al., 2010; DiGiulio, 2012; Healy et al., 2022). Although previous studies have continuously attempted to elucidate the microbial communities of neonatal gut (Schwiertz et al., 2003; Palmer et al., 2007; Korpela et al., 2018), the overall understanding of the gut microbiome in preterm infants cared for in the neonatal intensive care unit (NICU) remains inadequate because of a relatively scarce attempt at sample analysis and the small number of participants studied. For decades, meconium has been considered sterile (sterile womb hypothesis), but some studies using molecular techniques suggest that meconium contains a complex microbiota (*in-utero* colonization hypothesis) (Hansen et al., 2015; Koleva et al., 2015). Recently, noteworthy studies have demonstrated again that meconium does not have detectable microbiome taxa due to insufficient bacterial read, and too low concentrations of DNA extraction (Kennedy et al., 2021; Martí et al., 2021; Klopp et al., 2022).

Next-generation sequencing (NGS) has revolutionized the profiling of environmental and clinical microbial communities in recent years (Leggett et al., 2020). Currently, second-generation NGS platforms, such as Illumina (San Diego, CA, USA), have been used for clinical *in vitro* testing with US FDA approval. However, the high cost of Illumina platforms and their large spatial footprint can make their application in clinical settings challenging. Short-read NGS technology-based 16S rRNA analysis is limited to a maximum of ~450 bp of 16S rRNA hypervariable regions, namely V3–V4 and V4–V5 (Biol-Aquino et al., 2019; Fadeev et al., 2021), among others. Depending on the variability in 16S rRNA regions between phylogenetically closely related microbes, such innate limitations can cause difficulties in identifying different microbes at the species and strain levels and even for specific microbes that are practically identical in the target amplicon region. It has been reported that even subtle strain-level variations are vast in the case of closely related bee species (Ellegaard et al., 2020) and can cause pathogenicity within the human microbiome (Yan et al., 2020); short-read-based 16S rRNA analysis methods may overlook potential epidemiologic points of interest in some microbial analytical settings.

Oxford Nanopore Technologies (ONT) sequencing is an innovative third-generation long-read NGS method of single-molecule real-time (SMRT) sequencing. The MinION device by ONT (Oxford, United Kingdom), a pocket-sized sequencing platform capable of producing runs comparable to Illumina and PacBio (Leggett et al., 2020), represents an inexpensive and portable sequencing platform capable of producing long-read data. Using ONT sequencing is a cost-effective approach for bacterial identification at the species-level owing to its capital cost and the ability to sequence the full 16S rRNA gene region and more. Outbreak surveillance and characterization of low microbial biomass samples are required (Ashton et al., 2015; Greninger et al., 2015; Quick et al., 2016; Schmidt et al., 2016), as in NICU environments, which can be aided by the advantages of ONT sequencing. Given the methodological differences in the 16S rRNA target regions between Illumina (V3-V4) and ONT (V1-V9), their sequencing results and success rates can differ, especially at low DNA loads such as neonatal meconium.

This study aimed to compare two sequencing methods ONT MinION and Illumina in infants’ gut microbiome and to compare changes between term and preterm infants using ONT MinION. This is the first study to compare the gut microbiome of term and preterm infants using both sequencing methods and provides the first step toward using ONT platforms in NICU.

## Materials and methods

### Subjects and fecal sampling

We prospectively enrolled infants who were hospitalized in the newborn nursery room (NB) or NICU of Hanyang University Hospital, Seoul, Korea, from July 2021 until January 2022. The term infants (T) group was the control that was defined as ≥37 weeks of gestation and they did not receive intravenous antibiotics after birth. Infants with a gestational age of <37 weeks constituted the preterm group (P). Healthy P infants were hospitalized in the NICU and divided into two subgroups according to gestational age (GA): very preterm (VP, 28^+0^ ≤ GA ≤31^+6^ weeks) and moderate-to-late preterm (LP, 32^+0^ ≤ GA ≤ 36^+6^ weeks). All infants were enrolled in the study within 2 days after birth. Fecal samples were collected by a trained pediatrician during two periods: within 7 days of birth (T1, P1) and at 28 ± 7 days (T2, P2). Infants with major congenital anomalies or malformations, unstable conditions including septic shock, and necrotizing enterocolitis (NEC) were excluded from the study. The study was approved by the Institutional Review Board of Hanyang University Medical Center (No. 2021-03-017). Written informed consent was obtained from the parents of the infants and all methods were performed in accordance with standard human research ethics guidelines (Declaration of Helsinki).

**Figure 1:**
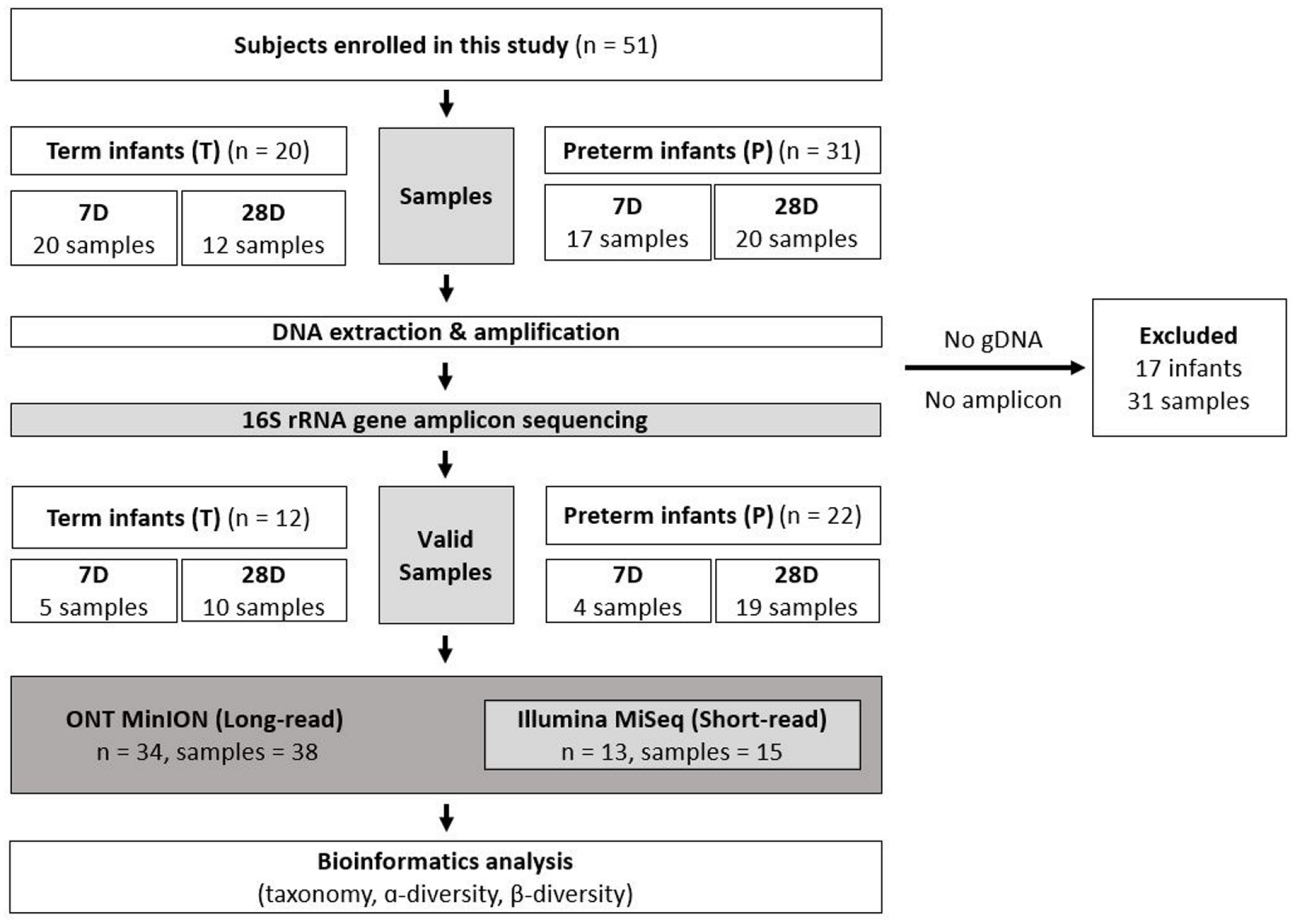
Sampling protocol and analysis method for participants. The subjects were grouped according to gestational age (37 weeks). Fecal samples were collected at 7 and 28 days after birth. ONT was performed on 38 fecal samples obtained from 34 infants. To compare the sequencing results, 15 overlapping samples from 13 infants were analyzed using Illumina. T, Term infants; P, Preterm infants; 7D, day 7 of life after birth; 28D, day 28 of life; DNA, Deoxyribonucleic Acid; rRNA, Ribosomal ribonucleic acid; ONT, Oxford Nanopore Technologies.

A total of 51 infants including preterm (*n* = 31) and term (*n* = 20) who were hospitalized in the NICU or nursery room were enrolled and 69 fecal samples (*T1* = 20, *T2* = 12; *P1* = 17, *P2* = 20) were collected from them. After DNA extraction, amplification, and sequencing, 31 fecal samples (*T1* = 15, *T2* = 2; *P1* = 13, *P2* = 1) from 17 infants (*T* = 8; *P* = 9) were excluded due to insufficient concentrations of gDNA or library amplicons. Using ONT, valid results from 38 fecal samples (*T1* = 5, *T2* = 10; *P1* = 4, *P2* = 19) from 34 infants (*T* = 12; *P* = 22) were obtained. Additionally, 15 overlapping samples (*T1* = 1, *T2* = 3; *P1* = 4, *P2* = 7) from 13 infants (*T* = 4; *P* = 9) were analyzed using Illumina to test the effectiveness of ONT sequencing (Figure 1).

### Clinical data

Clinical data were extracted from the medical records, at the time of fecal sample collection. Demographic characteristics included sex, delivery mode, birth weight, Apgar scores at 1 and 5 min, maternal gestational diabetes mellitus (GDM), pregnancy-induced hypertension (PIH), acute chorioamnionitis, use of antenatal steroids, and maternal antibiotic exposure. Information on after birth parameters, such as the use of antibiotics (within 48 h or on the day of sample collection), the day of invasive ventilator administration, and breastmilk exposure at the time of sample collection, was also collected.

### Fecal DNA extraction

Up to 1 g of feces from diapers was collected in a sterilized Cary-Blair aqueous solution tube using a sterilized microbiological sampling swab in the FecalSwab Sampling Kit (470 CE, Copan, Italy) and stored in a cryogenic freezer at −80°C. In cases where fecal sample collection was not feasible, feces were collected using a rectal swab. If it was difficult to store the samples immediately in the cryogenic freezer, they were first stored in the sample freezer at −20°C and transferred within a week to a cryogenic freezer for storage.

A DNeasy PowerSoil Pro Kit (QIAGEN, Hilden, Germany) was used for genomic DNA extraction from the microorganisms in the sample, according to the manufacturer’s instructions, except for the initial bead homogenization conditions. A bead homogenizer (Bead Ruptor Elite, OMNI International, Kennesaw, GA, USA), with bead-beating conditions of 1-min homogenization at 6 m/s, 5-min rest, with a total of 3-min homogenization time, using a ceramic bead mixture of diverse sizes was used. The sample input was 100–150 μL, and in case when the extraction was not processed properly, additional improvement measures were applied; the pellet from a 300–400 μL sample centrifuged at 15,000 *g* was used and the elution reagent was heated to 60–70°C before final DNA elution. The elution volume ranged from 50 to 100 μL, and slightly fewer amounts of elute were recovered for each sample. The concentration and purity of the extracted DNA were checked using a Nanodrop Lite spectrophotometer (Thermo Fisher Scientific, Waltham, USA), which ranged from 1.4 to 138.5 ng/μL (mean value 21.7 ng/μL) for dsDNA concentration and 1.68–2.08 (mean 1.89) for dsDNA A260/A280 ratio. The extracted gDNA samples were stored at −80°C for subsequent processing.

### 16S rRNA gene amplicon barcoding PCR and library preparation

#### ONT MinION 16S V1–V9 library preparation

The extracted gDNA was then prepared for prokaryotic metagenome sequencing using the 16S Barcoding Kit and 16S Barcoding Kit 0–24 (SQK-RAB204 and SQK-16S024, Oxford Nanopore Technologies, Oxford, UK), according to the manufacturer’s protocol, using 10 ng of the extracted gDNA per sample. The PCR reaction was performed on the full 16S hypervariable region (V1-V9) (Frank et al., 2008) with 27F forward primer (5′-ATCGCCTACCGTGAC – barcode – AGAGTTTGATCMTGGCTCAG – 3′) and 1492R reverse primer (5′ – ATCGCCTACCGTGAC – barcode – CGGTTACCTTGTTACGACTT – 3′) by injecting each multiplexing barcode included in the 16S Barcoding Kit 0–24 into 10 ng of each extracted DNA under the following conditions: initial 30 s denaturation at 98°C (Stage 1), 25 cycles of 10 s denaturation at 98°C, 30 s annealing at 55°C, 90 s extension at 65°C (Stage 2), and 5 min final extension at 65°C (Stage 3), with NEBNext^®^ Ultra^™^ II Q5^®^ Master Mix (New England Biolabs, Ipswich, MA, USA) as the PCR polymerase reagent mixture. The 16S V1–V9 amplicons were subsequently purified using Agencourt AMPure XP (Beckman Coulter, Brea, CA, USA) magnetic beads with a PCR reaction mix to magnetic bead ratio of 5:3 and washed twice with freshly prepared 70% ethanol. The final elution of purified DNA was performed by adding 12 μL of 10 mM Tris–HCl pH 8.0 with 50 mM NaCl, incubating for 2 min at room temperature, and recovering 10 μL of the elute from each tube. The concentration of each purified 16S V1–V9 amplicon was confirmed with an Invitrogen Qubit 4 fluorometer (Thermo Fisher Scientific, Waltham, MA, USA), and the samples were pooled with a total concentration of 100 fmol in 10 μL of the final library.

#### Illumina MiSeq 16S V3–V4 library preparation

Sequencing libraries were prepared according to Illumina 16S Metagenomic Sequencing Library protocols to amplify the V3 and V4 regions of the 16S rRNA gene. The input of 2 ng gDNA was PCR-amplified with 5x reaction buffer, 1 mM dNTP mix, 500 nM each of the universal F/R PCR primers, 5 pM each of the pRNA, mRNA oligo, and Herculase II fusion DNA polymerase (Agilent Technologies, Santa Clara, CA, USA). The cycle conditions for the 1st PCR were 3 min at 95°C for heat activation and 25 cycles of 30 s at 95°C, 10 s at 78°C, 60 s at 50°C, and 60 s at 72°C, followed by a 5-min final extension at 72°C. The universal primer pair with Illumina adapter overhang sequences used for amplification was as follows: V3-F:5′-TCGTCGGCAGCGTCAGATGTGTATAAGAGACAGCCTACGGGNGGCWGCAG-3′, V4-R:5′-GTCTCGTGGGCTCGGAGATGTGTATAAGAGACAGGACTACHVGGGTATCTAATCC-3′, based on the V3–V4 universal primers (Klindworth et al., 2013). The first PCR product was purified using AMPure XP beads (Beckman Coulter). Following purification, 2 μL of the purified eluate was again PCR-amplified for final library construction containing the index using the NexteraXT Indexed Primer. The cycle conditions for the second PCR were the same as those for the first PCR, except for the number of cycles which was 10. The PCR products were purified using AMPure XP beads. To verify the size of PCR-enriched fragments, the template size distribution was checked using a TapeStation D1000 Screen Tape (Agilent Technologies, Waldbronn, Germany).

### 16S rRNA gene amplicon sequencing

For the prepared ONT library sequencing, MinION Flow Cell (R9.4.1, FLO-MIN-106D, Oxford Nanopore Technologies, Oxford, UK) with MinKNOW software (v21.06.13, Oxford Nanopore Technologies) was utilized. FAST5 raw data were collected for up to 48 h for each run, simultaneously processing the FAST5 data to the FASTQ file using a GPU-based ont-guppy basecaller (v5.0.16, Oxford Nanopore Technologies) in super-accurate mode with barcode and adaptor trimming, until the number of passed reads for the barcode of the lowest value exceeded 100,000. As the sequencing capacity of MinION Flow Cells was much higher than the maximum of 24 samples that could be multiplexed with the SQK-16S024 barcoding kit, after reaching the desired throughput for a sequencing run, the MinION cells were washed with the Flow Cell Wash Kit (EXP-WSH004, Oxford Nanopore Technologies) after each run, stored, and reused until the pores were depleted. For the Illumina V3–V4 library, the MiSeq platform was used to sequence the index-attached V3–V4 amplicons under 300 × 2 paired-end read conditions using standard Illumina 16S Metagenomic Sequencing Library protocols to obtain FASTQ data of read count ranging from 121,470 to 609,040.

### Bioinformatics and statistical analysis

#### FASTQ quality control and processing

For ONT sequencing, after the sequencing run, the FASTQ files that passed the quality filter were concatenated into one file per sample. Subsequently, the *fastp* package (Chen et al., 2018) was used to filter out the reads further to remove contaminants and artifacts that hinder the downstream processing of the data. The remaining reads were subsampled with 50,000 reads per sample using the *seqtk* package for taxonomic analysis and 15,000 reads per sample for phylogenetic tree alignment under the available computational resources. The reliability of the subsampled read numbers was verified. Prior to subjecting the full dataset to taxonomic analysis, three random samples from the ONT sequencing were subsampled to 30,000 reads and 100,000 reads per sample to ensure adequate read depth was achieved. The results showed negligible differences of less than 0.1% in all taxonomic levels, showing that the read depth of 30,000 reads per sample was sufficient for detecting minor constituents of the 16S metagenome. For the main taxonomic analysis, 50,000 reads per sample were used to further ensure adequate read depth. For diversity analysis, the alpha rarefaction curves of various alpha diversity parameters were checked to verify the plateau of the curves.

Illumina sequencing data were obtained in the FASTQ format and demultiplexed by each barcode. Paired-end reads were merged, and chimeric reads were eliminated. All the obtained raw reads were subjected to taxonomic pipeline analysis, after the adapter and low-quality sequences of the reads were trimmed using Trimmomatic (v0.39).

#### Taxonomic analysis

For taxonomic analysis of the ONT data, the full MetONTIIME pipeline was designed for use with CPU-based ont-guppy basecalling of the FAST5 data, and the *MetONTIIME.sh* shell script file was utilized for taxonomic analysis of the GPU-basecalled FASTQ data obtained previously. To compare the relative abundance of each sample and their corresponding groups, the QIIME2 platform (v2022.2) (Bolyen et al., 2019) and MetONTIIME pipeline were used for downstream processing of FASTQ data. The SILVA ribosomal RNA gene reference database version 138.1 NR99 (Quast et al., 2013) with curation methods provided by the RESCRIPt package (Robeson et al., 2021) was used for read mapping. First, sequences containing five or more ambiguous bases were removed and differentially filtered among kingdoms by discarding reads under 900 bp for archaea and under 1,200 bp for bacteria. The filtered sequences were dereplicated further to create a consensus taxonomy, extracted with 27F-1492R primers to obtain the amplicon region, and finally dereplicated again from an optimized reference database for more efficient taxonomic analysis. The default parameters provided by the QIIME2 pipelines were utilized unless otherwise specified. The FASTQ reads were dereplicated using *vsearch* (Rognes et al., 2016) and clustered into *de novo* operational taxonomic units (OTUs) to avoid reference bias. The clustered OTUs were taxonomically classified using *vsearch* global alignment with an identity threshold of 85% and query coverage parameter of 0.8. The generated taxonomy file was tabulated for each taxonomic level of interest from the phylum to the species-level, and the relative abundance of the taxa was determined for comparison.

QIIME2 platform was likewise utilized for Illumina V3–V4 data; however, amplicon sequence variants (ASVs) were constructed with trimmed Illumina reads using DADA2 method (Callahan et al., 2016) as an advanced alternative available in short-reads compared to OUT generation, and then taxonomically classified with the reference database using 97% identity threshold (Pichler et al., 2018). The same SILVA 138.1 NR99 database was used, with V3-F and V4-R primer sequences being utilized to optimize the database for analysis. The generated taxonomy was tabulated from the phylum to the genus level, and the relative abundance was calculated.

#### Phylogenetic tree alignment and diversity analysis

For ONT reads, the subsampled FASTQ files were dereplicated and clustered *de novo* with the same parameters as in the taxonomic analysis, and the clustered OTUs were used for phylogenetic tree alignment using the *MAFFT* (Katoh et al., 2002) alignment program with the FFT-NS-2 alignment method along with *FastTree* (Price et al., 2010) software. Subsequently, using the generated rooted tree phylogeny, alpha diversity metrics and rarefaction curves of up to 15,000 reads were generated, and beta diversity analysis of Bray–Curtis dissimilarity and weighted/unweighted UniFrac distances (Lozupone et al., 2011) were ordinated using principal coordinate analysis (PCoA) with *q2-diversity* plugin in the QIIME2 platform.

#### Statistical analysis

All data were analyzed using Statistical System 9.4 (SAS Institute, Cary, NC, USA), and R statistical software version 4.0.3 (R Foundation). Clinical characteristics were carried out by analysis of variance, and linear correlation between ONT and Illumina was estimated as Pearson correlation. Significant differences between treatments were defined at *p* < 0.05, and *p* < 0.01 levels, and adjusted *P* (*Q* value) < 0.05 was considered as significant differences in diversity analysis.

## Results

### Study population

The T and P groups differed in gestational age (38.8 ± 0.9 vs. 32.3 ± 2.8, *p* < 0.001) and birth weight (3190 ± 229.4 vs. 1688 ± 600.2, *p* < 0.001), and delivery mode (*p* = 0.037). However, there were no differences in sex, delivery mode, the start of feeding day, or current breastfeeding. T, the control group was not exposed to antibiotics. However, according to the NICU guidelines of Hanyang University Hospital, most preterm infants are exposed to antibiotics. Except for GA and birth weight, there were no significant differences in the use of antibiotics, duration of antibiotics, delivery mode, and current breastfeeding between VP and LP groups (Supplementary Table 1). Baseline characteristics and infant outcomes are detailed in Table 1.

**Table 1:** Relevant clinical characteristics of infants whose gut microbiome was analyzed ^1, 2, 3^.

Characteristics	Term (*n* = 12)	Preterm (*n* = 22)	*p*
GA, weeks	38.8 ± 0.9	32.3 ± 2.8	<0.001
Birth weight, g	3190.0 ± 225.8	1688.6 ± 600.2	<0.001
Male, n	9 (75.0)	4 (72.2)	0.071
Apgar score			
1-min	8.3 ± 1.0	4.2 ± 2.1	<0.001
5-min	9.5 ± 0.6	7.0 ± 1.5	<0.001
C-section, n	9 (75.0)	22 (100)	0.037
Antibiotics exposure, n	0 (0.0)	20 (90.9)	
Breastmilk feeding, n	9 (75.0)	10 (45.5)	0.152
Hospitalization, days	4.9 ± 0.9	43.6 ± 24.9	<0.001

^1^GA, gestational age; C-section, cesarean section.

^2^Term infants were born at a GA ≥ 37 weeks. Antibiotic exposure refers to administering antibiotics to neonates in the first 48 h of life. The delivery mode was either cesarean section or vaginal delivery. Breastmilk feeding was defined as breast milk consumption at the time of sample collection.

^3^Data are presented as n (%) or mean (±SD), unless otherwise stated.

### Term vs. preterm infants (7  days and 28  days)

#### Phylum level

##### Firmicutes phylum predominated both T1 and P1 profiles

**Figure 2:**
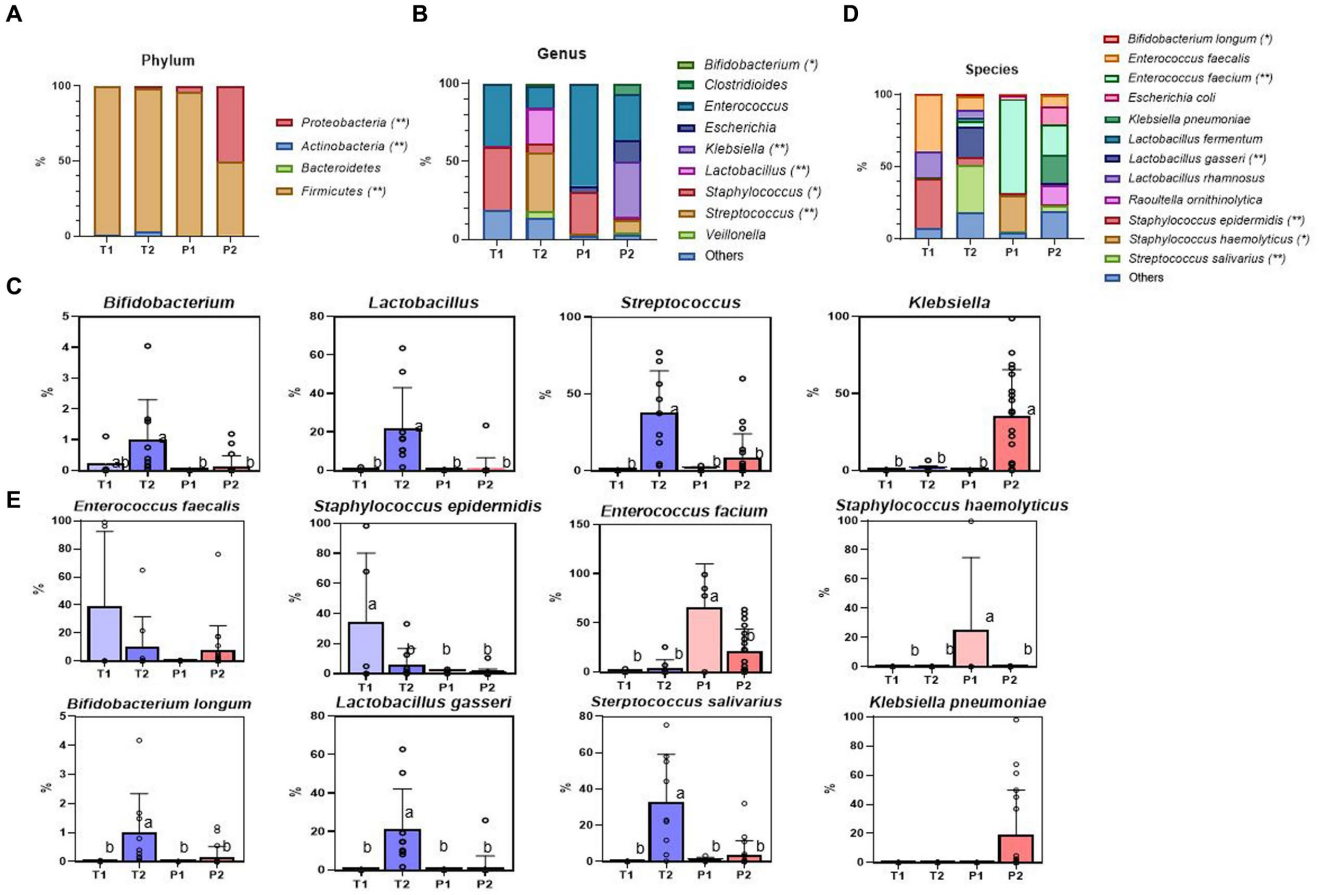
Relative abundance of the most dominant bacterial communities, analyzed by ONT. **(A)** Relative abundance at the phylum level. **(B)** Relative abundance at the genus level. **(C)** The specific bacterial population at the genus level (*Bifidobacterium*, *Lactobacillus*, *Streptococcus*, *Klebsiella*) **(D)** Relative abundance at the species-level. **(E)** The specific bacterial population at the species-level (*Enterococcus faecalis*, *Staphylococcus epidermidis*, *Enterococcus faecium, Staphylococcus haemolyticus*, *Bifidobacterium longum*, *Lactobacillus gasseri*, *Streptococcus salivarius*, *Klebsiella pneumoniae*). Treatment groups: T1, term group in period 1; T2, term group in period 2; P1, preterm group in period 1; P2, preterm group in period 2. *, ** indicate significant differences between groups (*p* < 0.05, and *p* < 0.01, respectively). ^a,b^ Mean values within a row having different superscript letters are significantly different (*p* < 0.05).

The dominant phylum identified in T1 was *Firmicutes* (99.05%), followed by *Actinobacteria* (0.68%), *Proteobacteria* (0.22%), and *Bacteroidetes* (0.05%) (Supplementary Table 2). In P1, *Firmicutes* was the most abundant (96.05%), followed by *Proteobacteria* (3.86%) and *Actinobacteria* (0.06%). No *Bacteroidetes* were present (Figure 2A).

##### Proteobacteria and *Firmicutes* dominated the P2 microbiome

Over time, *Proteobacteria* increased significantly in P2 (P1 vs. P2, *p* < 0.011), almost at the same level as *Firmicutes*. By contrast, *Actinobacteria* barely increased in abundance. There were statistically significant increases in the abundances of *Firmicutes*, *Proteobacteria*, and *Actinobacteria* (*p* < 0.001, *p* < 0.001, and *p* < 0.001, respectively) at 28 days (Figure 2A).

#### Genus level

##### Genus-level profiles showed different bacterial compositions in T1 vs. P1

Within the same *Firmicutes* phylum, *Staphylococcus* (40.33%) was the most abundant in T1, followed by *Enterococcus* (39.76%) and *Lactobacillus* (0.41%), whereas, in P1, *Enterococcus* (65.58%) was dominant, followed by *Staphylococcus* (27.01%) and *Lactobacillus* (0.09%) (Figure 2B).

##### Prematurity influenced the abundance of *Klebsiella*, *Streptococcus*, *Lactobacillus*, and *Bifidobacterium* genus not at 7 days but at 28 days

There were no significant differences between T and P at the genus level after 7 days of life. However, at 28 days, we observed a noticeable increase in the relative abundance of *Klebsiella*, a potential bacterial pathogen, (*p* = 0.002) in P2, and *Streptococcus* and beneficial *Bifidobacterium* and *Lactobacillus* in T2 (*p* = 0.001, *p* = 0.026, *p* < 0.001, respectively) (Figure 2C).

##### Genus-level profiles were different in T1 and T2

*Firmicutes* remained the most abundant phylum from T1 to T2. However, the dominant genera changed from *Staphylococcus* (40.33%), *Enterococcus* (39.76%), and *Lactobacillus* (0.41%) to *Streptococcus* (37.55%), *Lactobacillus* (22.07%), and *Enterococcus* (13.50%) (Supplementary Table 3).

### Profiling up to species-level through ONT application

#### There were differences at the species-level, even in the same genus (T1 vs. P1)

The T1 showed higher abundance of *Enterococcus faecalis* (*p* = 0.081) and *Staphylococcus epidermidis* (*p* = 0.004), whereas in P1, *Enterococcus faecium* (*p* < 0.001) and *Staphylococcus hemolyticus* (*p* = 0.034) were more abundant (Figures 2D,E).

#### Beneficial bacteria increased in T2, whereas pathogenic bacteria increased in P2

*Bifidobacterium longum, Lactobacillus gasseri,* and *Streptococcus salivarius* significantly increased in T2 (*p* = 0.017, *p* = 0.001, *p* < 0.001, respectively), whereas in P2, *Klebsiella pneumoniae* was increased, but there was no statistical difference (*p* = 0.096) (Supplementary Table 4).

### Diversity analysis

#### α-diversity

**Figure 3:**
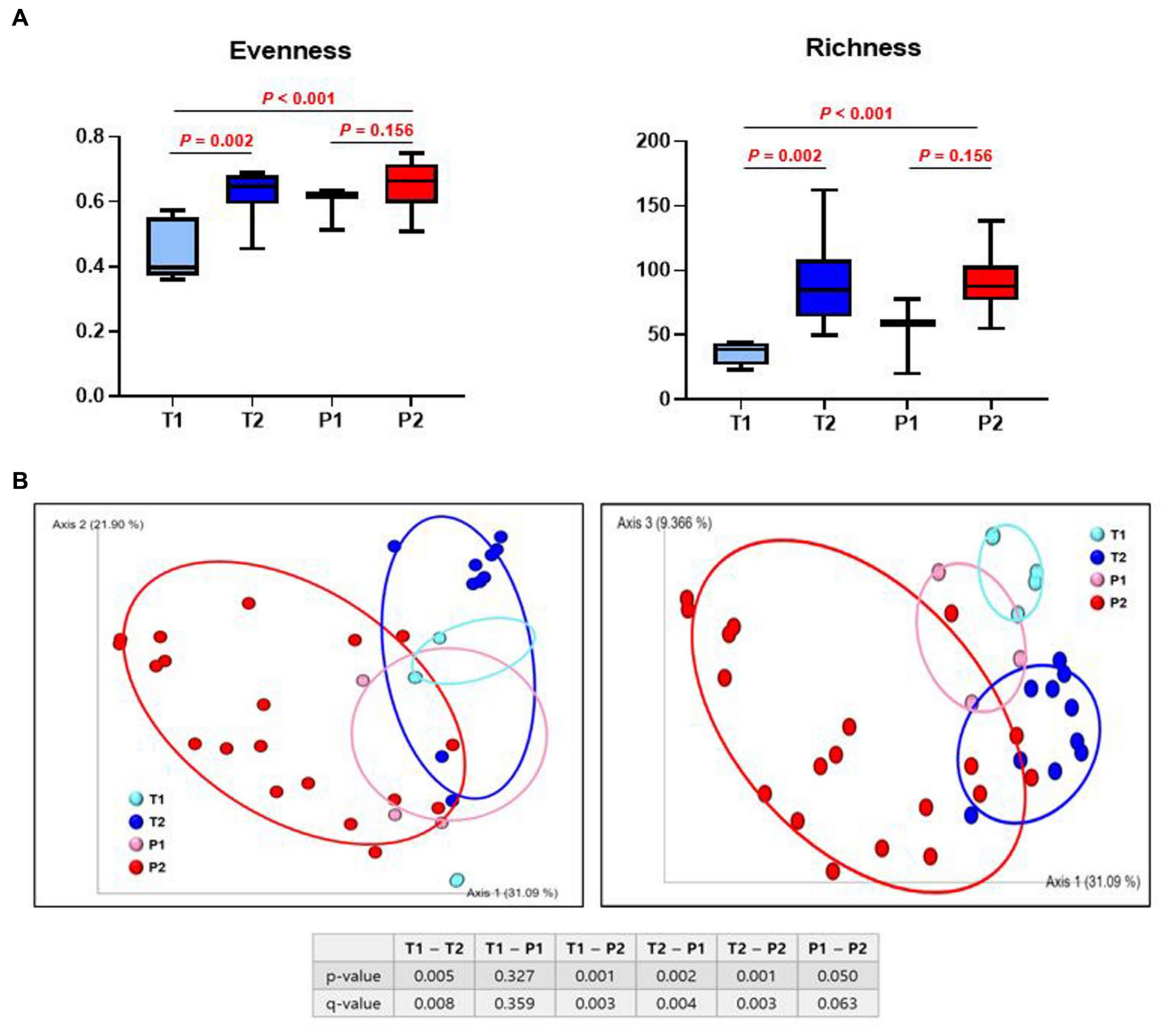
Difference in α- and β - diversity of gut microbiome between T and P. **(A)** Species evenness index (Pielou’s evenness index) and species richness index (Faith’s phylogenetic diversity). **(B)** Principal coordinate analysis (PCoA) ordination plots of microbial communities in T1, T2, P1, and P2 based on weighted UniFrac distance. *p*-value shows significant differences between groups. *q*-value shows FDR, adjusted *p*-value.

Bacterial α-diversity was measured using Pielou’s evenness index (microbial evenness) and Faith’s phylogenetic diversity index (species richness). A statistically significant increase was observed in both evenness and richness in T (T1 vs. T2; *p* < 0.002 and T1 vs. T2; *p* < 0.002, respectively), but P did not show difference in both evenness and richness (P1 vs. P2; *p* = 0.156 and P1 vs. P2; *p* = 0.156, respectively) (Figure 3A).

#### β-diversity

Subsequently, we evaluated the similarity of the gut microbiota (β-diversity) for both bacterial composition and phylogenetic relationships of the components. There was a significant difference in the β-diversity between T1 and T2 (*p* = 0.005), but no differences were observed between P1 and P2 (*p* = 0.050). Samples from T1 and P1 were dispersed and intermingled, suggesting that the bacterial composition of T1 was largely similar to that of P1 (*p* = 0.327). However, there was a statistically significant difference between T2 and P2 (*p* = 0.001) (Figure 3B).

### Microbiome differences in preterm infants with different gestational ages (VP vs. LP)

#### There were significant differences between VP and LP

**Figure 4:**
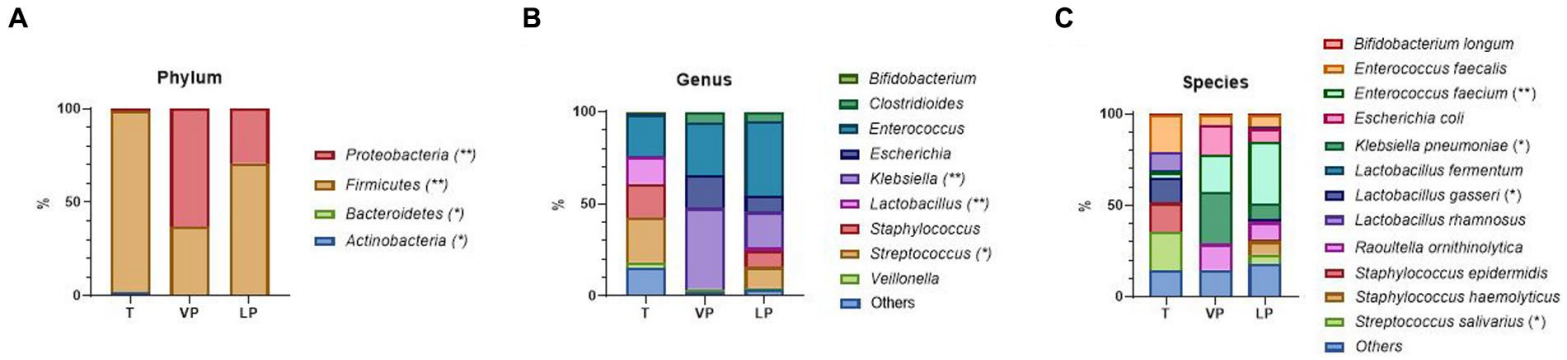
Difference in relative abundance of the most dominant bacterial communities between VL and LP, as analyzed by ONT. **(A)** Relative abundance at the phylum level. **(B)** Relative abundance at the genus level. **(C)** Relative abundance at the species-level. Treatment groups: T, term group; VP, very preterm infants; LP, moderate to late preterm infants. *, ** indicate significant differences between groups (*p* < 0.05, and *p* < 0.01, respectively).

To investigate the impact of the degree of prematurity on the gut microbiome of premature infants, we divided them into two groups (VP and LP). VP infants had the greatest abundance of *Klebsiella pneumoniae* (*Klebsiella* genus/*Proteobacteria* phylum) (*p* = 0.014), whereas LP, later GA premature infants, had the greatest abundance of *Enterococcus faecium* (*Enterococcus* genus/*Firmicutes* phylum) (*p* = 0.008) (Figures 4A–C). Across all time points, in T, *Lactobacillus gasseri* (*Lactobacillus* genus/*Firmicutes* phylum) (*p* = 0.023) and *Streptococcus salivarius* (*Streptococcus* genus/*Firmicutes* phylum) (*p* = 0.013) were significantly increased.

### Comparison of ONT vs. Illumina sequencing performance in infants

**Figure 5:**
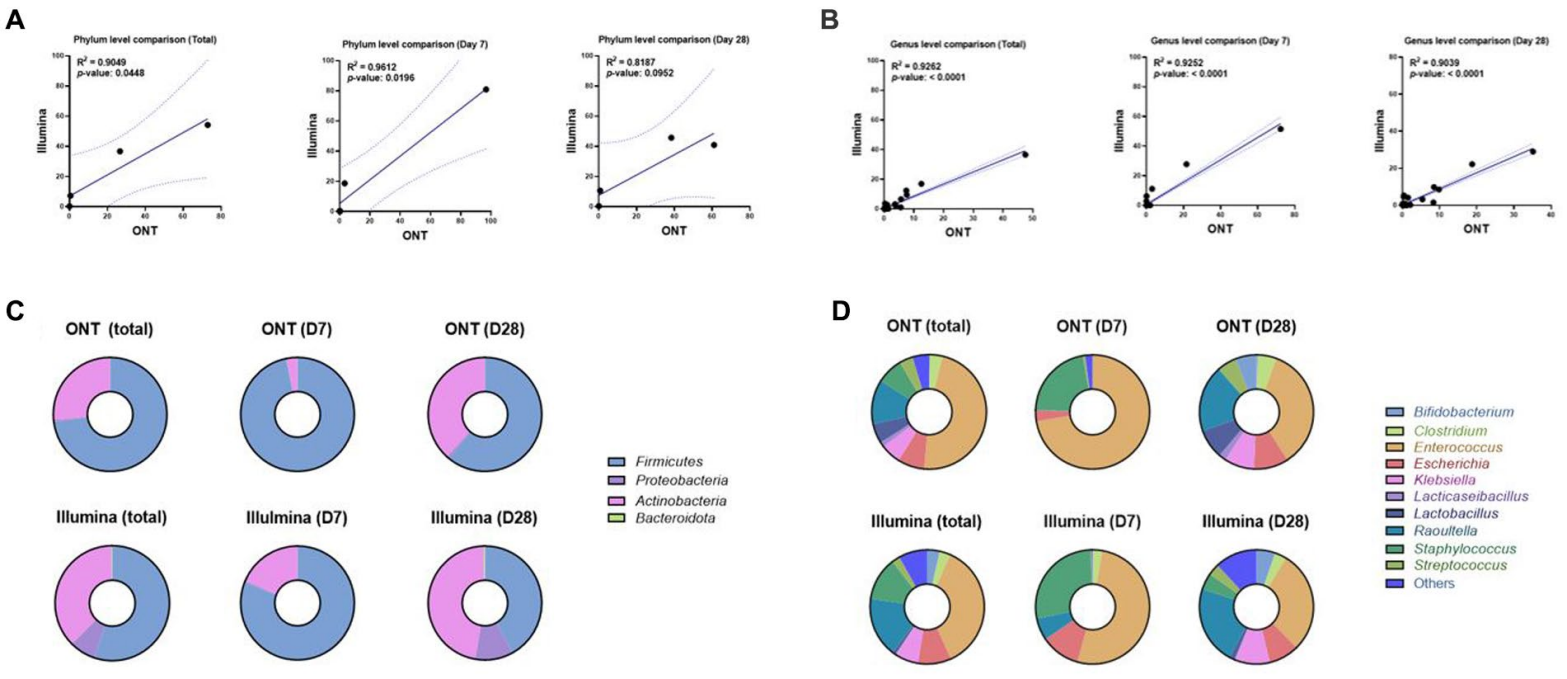
Correlation analysis between ONT and Illumina sequencing methods in phylum and genus level at D7 or D28. **(A)** Correlation plot of gut microbial abundance in phylum level of ONT (*x*-axis) and Illumina (*y*-axis) data at 7D or 28D (Pearson’s *r*^2^ = 0.905, Total; Pearson’s *r*^2^ = 0.961, D7; Pearson’s *r*^2^ = 0.819, 28D). The region on either side of the dotted line represents the 95% CIs. **(B)** Correlation plot of gut microbial abundance in genus level of ONT (*x*-axis) and Illumina (*y*-axis) data at 7D or 28D (Pearson’s *r*^2^ = 0.926, Total; Pearson’s *r*^2^ = 0.925, 7D; Pearson’s *r*^2^ = 0.904, 28D). The region on either side of the dotted line represents the 95% CIs. **(C)** Taxonomic profiles of gut microbiome abundance at 7D or 28D in phylum level. The top row corresponds to the results from ONT, and the bottom row shows the results from Illumina. **(D)** Taxonomic profiles of gut microbiome abundance at 7D or 28D in genus level. The top row corresponds to the results from ONT, and the bottom row shows the results from Illumina. 7D, day 7 of life after birth; 28D, day 28 of life; ONT, Oxford Nanopore Technologies.

We sequenced using Illumina for identical samples from 13 infants and compared them with ONT sequencing results up to the genus level. This approach showed that the ONT sequencing depth was sufficient to capture the bacterial diversity in the infants’ fecal samples. Taxonomic assignments using ONT versus Illumina sequencing data were largely consistent in both phylum (log-transformed Pearson’s *r*^2^ = 0.905) and genus (log-transformed Pearson’s *r*^2^ = 0.926) (Figures 5A,B). As well Firmicutes and Proteobacteria (Figure 5C), especially *Enterococcus*, *Escherichia*, *Klebsiella*, *Raoultella*, *Staphylococcus*, and *Streptococcus* were well consistent (Figure 5D).

Two sequencing reads correlated well, showing that the *Firmicutes* phylum was the most dominant, followed by *Proteobacteria* in T1 and P1, *Firmicutes* was still dominant in T2, and *Proteobacteria* abundance was increased in P2. However, the relative abundance of *Actinobacteria* (and the associated genus *Bifidobacterium*) was higher according to Illumina results, and it was also the same in T2 and P2 (T1 vs. T2; *p* = 0.073, P1 vs. P2; *p* = 0.099, respectively) (Supplementary Table 5). Although highly correlative, the total reads mapped for the ONT sequencing tended to exhibit greater read counts with a higher percentage of mapped reads compared to the results obtained using Illumina.

## Discussion

In this study, we evaluated the evolution of the gut microbiome during the first 28 days of life in T and P using ONT and Illumina sequencing combined with bioinformatics tools that could be used to gather clinically relevant data. ONT sequencing data were comparable in discriminatory power to Illumina data, allowing microbiome and abundance profiling and exploring species-specific profiles. Our data revealed a statistically significant increase of pathogenic bacteria (*Klebsiella pneumoniae, Enterococcus faecium*) in P; by contrast, few beneficial bacteria (*Lactobacillus gasseri, Bifidobacterium longum*) were found in this group. This difference was more evident at 28 days than at 7 days after birth.

This study demonstrated that both sequencing techniques performed comparably up to the genus level, but some bacterial proportions were different. There are several reasons for this difference. Firstly, Illumina sequencing generally exhibited more significant read counts with a lower percentage of mapped reads; however, this did not affect detection limits compared to ONT sequencing (Claesson et al., 2010; Stefan et al., 2022). This may explain the overexpression of *Actinobacteria*, which showed a low abundance in our study. Secondly, the principles of both sequencing methods are completely different (Claesson et al., 2010; Winand et al., 2019). While Illumina sequences the V3–V4 hypervariable regions of the 16 s rRNA gene (Klindworth et al., 2013) and yields >99% read accuracy using the sequencing-by-synthesis method, ONT sequences the whole hypervariable regions of 16S rRNA genes from V1 to V9 using the nanopore (pore-forming protein) method and yields around 91–92% read accuracy (R9.4.1 nanopore). The advantages and disadvantages of each method are described in detail (Supplementary Table 6). The lower read accuracy of ONT might require the read depth to be higher and the quality control of the produced reads to be more thorough to mitigate the erroneous reads that may cause identifying a same DNA sequence as different sequences (Claesson et al., 2010; Stefan et al., 2022). Therefore, the misclassification of *Klebsiella oxytoca* as *Enterobacter* can occur even if the same reference is used for taxonomy (Stefan et al., 2022). Thirdly, this difference can be seen in the case of low-complexity samples such as infant feces (Winand et al., 2019).

Despite the current limitations, the expected advantages of ONT sequencing are as follows; (1) it provides near real-time analysis of DNA sequences when coupled with cloud-based software, which can save time by identifying antibiotic-resistant bacteria, (2) it can enable the rapid selection of appropriate antibiotics in a clinical setting such as NICU (Ashton et al., 2015; Leggett et al., 2020; Stefan et al., 2022), and (3) in comparison to short-read data such as Illumina, bacterial identification at the species-level works well. At phylum level, the high accuracy of Illumina reads demonstrated superb classification performance, being able to use higher percent identity in the alignment compared to ONT data. However, it was comparably similar in genus level analysis, and in species-level, only ONT data was utilizable as majority of V3–V4 Illumina reads were not assigned. The specific details of unassigned portion of the reads are provided in detail (Supplementary Table 7).

The empirical differences in taxonomic analysis based on Illumina and ONT reads are based on their innate differences in resolving power in case of 16S rRNA gene-based analysis. The error rate of ONT sequencing, for the R9.4.1 pores and the corresponding chemistry, typically averages in the range of Phred Q-score of 10–15, which corresponds to 90.0 to 96.8% read accuracy. Thus, the lower percent identity threshold of 85% was implemented, as higher parameter has a possibility to erroneously assign the reads. Nevertheless, we observed that the ONT sequencing reads were able to be discerned down to species-level. This phenomenon can be described as follows. In terms of theoretical background, typically ~97% identity is applied for high-accuracy short-reads (i.e., Illumina, Ion Torrent, etc.), as they have very high read accuracy, and because their read length is short, which usually spans just one or two hypervariable regions of the 16S rRNA gene. Even with the longest short-read target region of V3-V4 (341F & 805R), which is a popular universal primer set employed for prokaryotic microbiome analysis, the amplicon size is 460 bp. Here, with 97% identity, around 445 bp is used to resolve the taxonomy. On the other hand, ONT-based full 16S hypervariable region analysis (V1-V9) has an amplicon size of 1,465 bp, and with an identity threshold of 85%, around 1,170 bp is utilized to resolve the taxonomy. This requires the percent identity threshold to be lower than the read accuracy, thus the parameter of 85% was used for the taxonomic analysis of the ONT reads. Nevertheless, even with the lower percent identity threshold, the ONT reads have around 2.6 times higher amplicon templates to resolve the read (445 bp vs. 1,170 bp). With the V3-V4 amplicon reads successfully resolving most of the reads down to genus level, we observed that V1-V9 amplicon reads, even when the percent identity of 85%, were able to be effectively discerned down to species-level, which was reported to be achievable with the older version of nanopores (Benítez-Páez et al., 2016; Shin et al., 2016), and now being more reliable with the updated pore version of R9.4.1 with higher read accuracy. The species-level analysis using ONT reads on 16S rRNA gene full-region has been continuously being reported with successful results (Nygaard et al., 2020; Huggins et al., 2022; Rozas et al., 2022), also in clinical targets (Komiya et al., 2022), even bringing forth the developments of ONT-dedicated species-level analysis workflows such as NanoCLUST (Rodríguez-Pérez et al., 2021).

Generally, the microbiome of healthy T and P infants after birth consists of four phyla: *Firmicutes*, *Proteobacteria*, *Actinobacteria,* and *Bacteroidetes* (Arboleya et al., 2015). Bacterial colonization in the gut changes rapidly after birth, especially for aerotolerant microbes (Unger et al., 2015). Infants have a primarily aerobic gastrointestinal tract, which promotes the appearance of facultative anaerobic bacteria, such as *Firmicutes* (*Enterococcus*, *Staphylococcus*, *Streptococcus*) and *Proteobacteria* (*Enterobacter*, *Escherichia*) (Senn et al., 2020). P infants show low diversity with increased colonization of potentially pathogenic bacteria such as Proteobacteria (*Klebsiella* and *Enterococcus*) and reduced levels of strict anaerobes such as *Bifidobacterium*, *Bacteroides*, and *Atopobium* (Rinninella et al., 2019). Consistent with the results of previous studies, our study also identified that T and P infants’ guts were dominated by Firmicutes (Arboleya et al., 2012, 2015), and P infants had higher Proteobacteria and fewer Actinobacteria at the phylum level (Tanaka et al., 2009; Fouhy et al., 2012). It also showed that preterm birth results in inadequate bacterial colonization for 28 days.

At the genus level, P infants had a significantly low abundance of protective commensal anaerobic bacteria such as *Bifidobacterium* and *Lactobacillus*. As *Bifidobacterium* has been associated with the expression of inflammatory response genes and the stimulation of genes that promote the integrity of the mucosal barrier, the risk of NEC in preterm infants increases with a decline in *Bifidobacterium*, resulting in an exaggerated inflammatory response (Esaiassen et al., 2017). Lactic acid bacteria such as *Lactobacillus gasseri* elicit various health benefits through their antimicrobial activity, bacteriocin production, and immunomodulation of innate and adaptive systems (Rodrigues da Cunha et al., 2012; Selle and Klaenhammer, 2013). In this study, over time, the abundance of *Bifidobacterium* and *Lactobacillus* increased in T infants but was significantly lower than that in P infants. Furthermore, P infants had excessive growth of potentially pathogenic bacteria, such as *Klebsiella* and *Enterococcus* over time. This finding is consistent with the results of previous studies (Tanaka et al., 2009; Zhu et al., 2017; Zou et al., 2018; Lu et al., 2020; Chang et al., 2021).

This study showed a significant increase in *Klebsiella* in P infants within 28 days, and among the *Klebsiella*, *K. pneumoniae* was dominant at the species-level. *Klebsiella* is a gram-negative bacterium, and one of the most common species is *K. pneumoniae*. *Klebsiella* is important in P infants, because *Klebsiella* overgrowth in the gut is highly associated with brain damage in EP infants and also associated with NEC, nosocomial infections, and late-onset sepsis (Carlisle et al., 2011; Zhuang et al., 2019; Chen et al., 2020; Paveglio et al., 2020; Ma et al., 2021; Seki et al., 2021). Gestational age remains a major determinant of gut microbiota colonization (Rinninella et al., 2019) and immunity. Younger individuals have a more immature immune system and a higher intestinal mucosal permeability (Sevastiadou et al., 2011; Strunk et al., 2011; Arboleya et al., 2015; Ananth et al., 2018). Compared with the gut microbiota of T infants, that of P infants is always characterized by decreased microbial diversity (Henderickx et al., 2019), but in our study, there was no difference in the evenness and richness of the microbiota in T and P infants. T showed significantly higher evenness and richness of gut microbiota over time, but there was no difference in P, which correlates with the results of previous studies (Arboleya et al., 2015; Chernikova et al., 2018; Chang et al., 2021; Kim et al., 2021).

Many studies have shown that the use of antibiotics reduces diversity and leads to dysbiosis of the microbiome. Previous studies have shown a decrease in *Actinobacteria* (including *Bifidobacteria*), and *Bacteroidetes*. In contrast, an increase was observed in *Proteobacteria* (including *Klebsiella* and *Escherichia*), and *Enterococcus* (Arboleya et al., 2015; Lu et al., 2020). Our study also showed consistent results with previous studies with decreased *Bifidobacteria* and increased *Klebsiella*. However, in this study, most preterm infants were exposed to antibiotics after birth, so it is difficult to know exactly whether the above results are due to antibiotics or gestational age. Other recent studies showed different results. Kim et al. (2021) showed that *Actinobacteria* were rather more abundant in the antibiotic-treated group. Moreover, the type, duration of usage, and combination of antibiotics may be more important factors than antibiotics use (Zhu et al., 2017).

Gut microbiome is affected by delivery mode. Cesarean section delayed the establishment of gut microbiota such as *Bifidobacteria* and *Bacteroidetes* (Kim et al., 2020). However, most high-risk neonates such as preterm infants are very often placed in situations where they were inevitably forced to be delivered by cesarean section to improve their survival (Ananth and Vintzileos, 2011). Consequently, we could not adjust this factor between two groups. Additionally, some studies have shown that the mode of delivery could influence the first colonizing microbes of the gut, but has less effect on the gut microbiome with time (Chu et al., 2017). La Rosa et al. (2014) showed preterm infants with a patterned progression of the gut bacterial community that is only minimally influenced by delivery modes, antibiotics, or feeds.

This study’s strength is in comparing the gut microbiome of P and T infants at two different time points using ONT and Illumina sequencing. Although there have been studies comparing the gut microbiome of T and P infants using Illumina (Chernikova et al., 2018; Jia et al., 2022), and studies identifying the gut microbiome of P infants using ONT (Ashton et al., 2015; Leggett et al., 2020), to our knowledge, this is the first study of gut microbiome comparison between T and P infants using ONT and Illumina together.

Nonetheless, this study has some limitations that need to be addressed in further studies. First, the number of samples is small, and there are missing data due to gDNA extraction failure or amplicons generation from 7-day samples. Some meconium 7-day samples did not show detectable microbiomes (Kennedy et al., 2021), which may be associated due to insufficient microbiota (Klopp et al., 2022). Moreover, Illumina sequencing platform was utilized for some 7-day samples, while other samples were analyzed on ONT. The differences in detection rates may have resulted from the innate differences of the 16S rRNA gene target regions of the two universal primer sets used in Illumina (341F-805R for V3-V4 region) and ONT (27F-1492R for V1-V9 region), as previously reported before the sample target region sets (Strube, 2021) and others such as V3-V4 to V4-V5 (Biol-Aquino et al., 2019; Fadeev et al., 2021) and V4-V5 to V5-V8 (McNichol et al., 2021) among many others. Therefore, the data not shown in our analysis are not missing at random, which can lead to comparison bias. In the future, finding from the above study can be helpful in designing amplicon sequencing strategies for 16S rRNA gene variable region. Finally, most preterm infants were given prophylactic antibiotics and delivered with Cesarean section, the condition of which has presumably affected the lower microbial abundance in the collected samples.

## Conclusion

Our results demonstrate for the first time that gestational age affects the evolution of gut microbiota in terms of composition and diversities, using ONT and Illumina together. The ONT sequencing was shown to have a high correlation with Illumina sequencing and may prove useful in clinical settings, particularly for infants’ microbial analysis and tailored intervention.

## Data availability statement

The data presented in the study are deposited in the NCBI Sequence Read Archive repository, accession number PRJNA871395.

## Ethics statement

The studies involving human participants were reviewed and approved by the Institutional Review Board of Hanyang University Medical Center (No. 2021-03-017). Written informed consent to participate in this study was provided by the participants’ legal guardian/next of kin.

## Author contributions

H-KP and B-HJ conceptualized, designed, and supervised the study. HK, M-JK, and JYP were involved in the data collection and initial statistical analysis. TC and JK performed the computational analyses, developed the algorithms, and interpreted the data. TC, JK, and H-KP wrote and edited the manuscript. All authors have reviewed and approved the revised manuscript.

## Funding

This research was funded by the Hanyang University (MEB) Global Center for Developmental Disorders (HY-202100000002865), Bio & Medical Technology Development Program of the National Research Foundation of Korea (NRF-2019M3E5D1A01069363).

## Conflict of interest

The authors declare that the research was conducted in the absence of any commercial or financial relationships that could be construed as a potential conflict of interest.

## Publisher’s note

All claims expressed in this article are solely those of the authors and do not necessarily represent those of their affiliated organizations, or those of the publisher, the editors and the reviewers. Any product that may be evaluated in this article, or claim that may be made by its manufacturer, is not guaranteed or endorsed by the publisher.

## Abbreviations

ONT: Oxford Nanopore Technologies
NICU: neonatal intensive care unit
NGS: next generation sequencing
T: term infants
P: preterm infants
GA: gestational age
VP: very preterm infants
LP: moderate to late preterm
NEC: necrotizing enterocolitis
GDM: gestational diabetes mellitus
PIH: pregnancy-induced hypertension
OTUs: operational taxonomic units
ASVs: amplicon sequence variants
