# Supplementary material for: Gut microbiome profiling of neonates using Nanopore MinION and Illumina MiSeq sequencing
Source: Front Microbiol. 2023 May 15;14:1148466. doi: 10.3389/fmicb.2023.1148466 (PMC10225602; doi:10.3389/fmicb.2023.1148466)
Supplement: Supplementary file 1 [file Data_Sheet_2.PDF]

Supplements

Supplementary Table 1. Clinical characteristics of preterm infants[1), 2), 3)]

**Characteristics**	**Very preterm (n=11)**	**Moderate to late preterm (n=11)**	***P***
GA, weeks	29.9 ± 1.2	34.7 ± 1.5	*< 0.001*
Birth weight, g	1194.5 ± 282.6	2142.7 ± 437.1	*< 0.001*
Male, n (%)	3 (27.3)	5 (45.5)	*0.659*
Apgar score			
1-min	4.3 ± 1.9	4.2 ± 2.3	*0.856*
5-min	6.7 ± 1.4	7.2 ± 1.5	*0.577*
C/sec, n	11 (100)	11 (100)	*1.000*
Antibiotics exposure, n	11 (100)	9 (81.8)	*0.476*
Duration of Antibiotics, days	4.2 ± 1.2	5.2 ± 2.4	*0.457*
Breastmilk feeding, n	5 (45.5)	4 (36.4)	*0.080*
Hospitalization, days	63.4 ± 12.2	21.4 ± 9.8	*< 0.001*

[1)]Abbreviations: GA, gestational age; C-section, cesarean section

[2)]Very preterm infants born at 28^{+0} ≤ GA ≤31^{+6} weeks. Moderate to late preterm infants were born at 32^{+0} ≤ GA ≤ 36^{+6} weeks. Antibiotic exposure refers to administering antibiotics to neonates in the first 48 h of life. The delivery mode was either cesarean section or vaginal delivery. Breastmilk feeding was defined as breast milk consumption at the time of sample collection.

[3)]Data are presented as n (%) or mean (±SD), unless otherwise stated.

Supplementary Table 2. Relative abundance differences between the term and preterm infant groups at phylum level[1)]

	T1	T2	P1	P2	*P*
Firmicutes, %	99.05[a]	95.54[a]	96.05[a]	49.12[b]	0.000
Proteobacteria, %	0.22[b]	1.63[b]	3.89[b]	50.51[a]	0.000
Actinobacteria, %	0.68[b]	2.80[a]	0.06[b]	0.37[b]	0.002
Bacteroidota, %	0.05	0.02	0.00	0.00	0.061

[1)]Abbreviations: T1, term-infant on day 7 after birth; T2, term-infant on day 28 after birth; P1, preterm-infant on day 7 after birth; P2, preterm-infant on day 28 after birth.

[a,b]Mean values within a row with different superscript letters are significantly different ($P < 0.05$).

Supplementary Table 3. Relative abundance differences between the term and preterm infant groups at genus level[1)]

	T1	T2	P1	P2	*P*
Bifidobacterium, %	0.23[ab]	1.00[a]	0.00[b]	0.14[b]	0.026
Clostridioides, %	0.00	0.87	0.04	6.52	0.079
Enterococcus, %	39.76	13.50	65.58	29.66	0.083
Escherichia, %	0.01	0.12	3.85	13.75	0.245
Klebsiella, %	0.15[b]	0.85[b]	0.02[b]	35.26[a]	0.001
Lactobacillus, %	0.41[b]	22.07[a]	0.09[b]	1.23[a]	0.000
Staphylococcus, %	40.33[a]	5.98[b]	27.01[a]	0.87[b]	0.012
Streptococcus, %	0.14[a]	37.55[a]	0.85[b]	8.30[b]	0.001
Veillonella, %	0.10	4.43	0.00	0.97	0.334

[1)]Abbreviations: T1, term-infant on day 7 after birth; T2, term-infant on day 28 after birth; P1, preterm-infant on day 7 after birth; P2, preterm-infant on day 28 after birth.

[a,b]Mean values within a row with different superscript letters are significantly different ($P < 0.05$).

Supplementary Table 4. Relative abundance differences in the 16 most abundant species between term and preterm infants[1)]

	T1	T2	P1	P2	*P*
Bifidobacterium longum, %	0.01[b]	1.02[a]	0.00[b]	0.15[b]	0.017
Clostridioides diffcile, %	0.00	0.00	0.04	2.44	0.306
Enterococcus faecalis, %	39.12	9.92	0.19	7.70	0.081
Enterococcus faecium, %	0.67[b]	3.84[b]	65.41[a]	20.93[b]	0.000
Escherichia coli, %	0.01	0.06	2.91	12.43	0.300
Finegoldia magna, %	0.00	1.96	0.00	0.68	0.457
Klebsiella pneumoniae, %	0.00	0.05	0.02	19.43	0.096
Lactobacillus fermentum, %	0.01	2.35	0.00	0.58	0.265
Lactobacillus gasseri, %	0.03[b]	21.24[a]	0.08[b]	1.36[b]	0.001
Lactobacillus rhamnosus, %	18.09	4.76	0.00	0.19	0.114
Raoultella ornithinolytica, %	0.08	0.05	0.00	13.54	0.144
Staphylococcus capitis, %	5.95	0.01	0.00	0.00	0.088
Staphylococcus epidermidis, %	34.26[a]	5.83[b]	1.26[b]	0.75[b]	0.004
Staphylococcus haemolyticus, %	0.06[b]	0.00[b]	24.93[a]	0.04[b]	0.034
Streptococcus salivarius, %	0.06[b]	32.58[a]	0.82[b]	3.48[b]	0.000
Veillonella atypica, %	0.04	3.78	0.00	0.00	0.165

[1)]Abbreviations: T1, term-infant on day 7 after birth; T2, term-infant on day 28 after birth; P1, preterm-infant on day 7 after birth; P2, preterm-infant on day 28 after birth.

[a,b]Mean values within a row with different superscript letters are significantly different ($P < 0.05$).

Supplementary Table 5. Intestinal microbial genus population of the term and preterm infants after birth, measured using ONT long-read and Illumina short-read sequencing techniques [1), 2)]

	Total (n = 15)		Term (n = 4)		Preterm (n = 11)	
	ONT	Illumina	ONT	Illumina	ONT	Illumina
**Genus level, %**						
Acidibacter	0.00	0.22	0.00	0.61	0.00	0.11
Anaerococcus	0.87	0.01	1.95	0.00	0.36	0.00
Bacillus	0.40	0.78	0.00	0.52	0.59	0.18
Bacteroides	0.00	0.37	0.00	0.00	0.00	0.29
Bifidobacterium	0.26	8.63	0.47	13.20	0.15	3.18
Blautia	0.00	0.17	0.00	0.00	0.00	0.08
Citrobacter	0.01	0.00	0.00	0.00	0.01	0.00
Clostridioides	0.88	3.04	0.00	0.00	1.29	3.62
Clostridium	2.76	2.92	0.98	0.00	3.61	3.32
Corynebacterium	0.06	0.26	0.15	0.00	0.01	0.00
Coxiella	0.00	0.00	0.00	0.51	0.00	0.14
Cronobacter	0.10	0.00	0.00	0.00	0.14	0.00
Cutibacterium	0.20	0.10	0.45	0.00	0.08	0.00
Dermabacter	0.00	0.01	0.00	0.00	0.00	0.00
Eggerthella	0.00	0.00	0.00	0.00	0.00	0.00
Enterobacter	0.59	1.61	0.01	0.00	0.86	3.99
Enterococcus	29.68	21.97	31.08	39.06	29.02	22.40
Erythrobacter	0.00	0.10	0.00	0.19	0.00	0.06
Escherichia	5.08	5.64	0.02	0.33	7.46	6.85
Haemophilus	0.00	0.45	0.00	0.00	0.00	0.29
Flavobacterium	0.00	0.00	0.00	0.00	0.00	0.01
Finegoldia	1.16	0.04	2.03	0.00	0.75	0.00
Klebsiella	9.79	9.22	0.89	0.74	13.98	12.63
Kluyvera	0.03	0.00	0.00	0.00	0.04	0.17
Lacticaseibacillus	1.37	0.10	3.83	0.53	0.20	0.00
Lactobacillus	5.35	0.49	13.76	2.58	1.39	0.26
Lactococcus	0.00	0.18	0.00	0.00	0.00	0.14
Limosilactobacillus	1.01	0.13	2.02	0.50	0.54	0.00
Luteimonas	0.00	0.18	0.00	0.00	0.00	0.10
Neobacillus	0.00	0.00	0.00	2.09	0.00	0.39
Nitrosospira	0.00	0.00	0.00	0.00	0.00	0.10
Ohtaekwangia	0.00	0.04	0.00	0.27	0.00	0.07
Paraburkholderia	0.00	0.00	0.00	0.52	0.00	0.24
Parabacteroides	0.00	0.02	0.00	0.00	0.00	0.00
Peptoniphilus	0.13	0.02	0.32	0.00	0.04	0.00
Peribacillus	0.00	0.00	0.00	0.17	0.00	0.00
Phocaeicola	0.00	0.00	0.00	0.00	0.00	0.05
Prevotella	0.00	0.02	0.00	0.00	0.00	0.06
Raoultella	18.08	16.10	0.12	0.19	26.54	19.52
Rhodococcus	0.00	1.39	0.00	2.40	0.00	0.60
Rothia	0.05	0.35	0.05	0.00	0.05	0.17
Rummeliibacillus	0.00	0.01	0.00	0.00	0.00	0.10
Sphingomonas	0.00	0.14	0.00	0.21	0.00	0.11
Salmonella	0.02	0.00	0.00	0.00	0.02	0.00
Serratia	0.11	0.00	0.00	0.00	0.16	0.00
Staphylococcus	12.82	9.04	1.61	14.89	18.09	10.78
Streptococcus	12.72	2.41	6.44	5.58	15.68	1.86

Ureaplasma	0.00	0.85	0.00	0.00	0.00	1.46
Veillonella	0.44	2.08	0.12	0.00	0.58	1.43
Vicinamibacter	0.00	0.21	0.00	0.88	0.00	1.00

[1)]Abbreviation: ONT, Oxford Nanopore Technologies

[2)] Term infants were born at a GA ≥ 37 weeks. Preterm infants were born at GA < 37 weeks.

Supplementary Table 6. The comparison of Nanopore and Illumina methods

	**Nanopore (ONT)**	**Illumina**
Country of origin	UK	USA
Instrument	MinION (+ Flongle), GridION, PromethION	iSeq, MiniSeq, MiSeq, NextSeq, NovaSeq
Release date	2015 (MinION)	2011 (MiSeq)
Type	- Long-read sequencing<br>- Nanopore (pore-forming protein)<br>- Electric current reading	- Short-read sequencing<br>- Sequencing-by-synthesis<br>- Fluorescence imaging
Advantages	- Long read length (>10,000 bp)<br>- Can sequence the full 16S rRNA hypervariable region (V1–V9) and longer<br>- Low instrument cost[1]<br>- Rapidly improving technology (Q20+, 99.2% modal accuracy for Kit 14 chemistry with R10.4.1 nanopore)[2]<br>- Higher mobility (palm sized, MinION)[1]	- Very high accuracy (Q30+, >99%)[1]<br>- Established technology (Ready-to-use bioinformatic pipelines)[3]<br>- Lower computational requirements for metagenomic applications due to existing optimized pipelines
Disadvantages	- Moderate accuracy (Q10+, 90~98%) (R9.4.1 nanopore)[1]<br>- Less optimal for applications that favor higher read accuracy (e.g., SNV, SNP)[4]<br>- Lower scalability[5]	- Short read length (<500 bp)[1]<br>- Can only sequence partial hypervariable regions in the 16S rRNA gene due to the short read length (e.g., V1–V2, V3–V4)<br>- Less mobility (Tabletop minimum)
Research in infants	Leggett, R.M., et al.[6]<br>Datta, M. S., et al.[12]<br>Dobbler, P. T., et al.[13]	Gibson, M.K., et al.[7]<br>Alcon-Giner, C., et al.[8]<br>Aguilar-Lopez M. et al.[9]<br>Jia, Q. et al.[10]<br>Korpela, K., et al.[11]

Supplementary Table 7. Unassigned reads for both sequencing methods at each target taxonomic level.

Rank	Parameter	ONT (V1–V9) (%)	Illumina (V3–V4) (%)
Phylum	Min	0.39	0.00
	Max	10.07	0.07
	Average	3.22	0.00
	SD	2.54	0.02
Genus	Min	0.39	0.00
	Max	11.28	29.32
	Average	3.50	2.49
	SD	2.67	7.25
Species	Min	0.74	6.14
	Max	17.69	100.00
	Average	6.29	73.11
	SD	4.12	26.23

Abbreviations: ONT, Oxford Nanopore Technologies; Min, Minimum; Max, Maximum; SD, Standard deviation.

Supplementary References

1. Kerkhof, L.J., et al. Is Oxford Nanopore sequencing ready for analyzing complex microbiomes? *FEMS Microbiol Ecol*. **97**, (2021).
2. Sereika, M., et al. Oxford Nanopore R10.4 long-read sequencing enables the generation of near-finished bacterial genomes from pure cultures and metagenomes without short-read or reference polishing*. Nat. Methods.* 2022. **19**, 823 (2022).
3. Klindworth, A., et al. Evaluation of general 16S ribosomal RNA gene PCR primers for classical and next-generation sequencing-based diversity studies*. Nucleic Acids Res*. **41**, (2013).
4. Lemay, M. A., et al. Combined use of Oxford Nanopore and Illumina sequencing yields insights into soybean structural variation biology. *BMC Biol*. **20**, (2020).
5. Amarasinghe, S.L., et al. Opportunities and challenges in long-read sequencing data analysis. *Genome Biol*. **21**, (2020).
6. Leggett, R.M., et al. Rapid MinION profiling of preterm microbiota and antimicrobial-resistant pathogens. *Nat Microbiol*. **5**, 430-442 (2020).
7. Gibson, M.K., et al. Developmental dynamics of the preterm infant gut microbiota and antibiotic resistome. *Nat Microbiol.* **1**, (2016)
8. Alcon-Giner, C., et al. Microbiota Supplementation with Bifidobacterium and Lactobacillus Modifies the Preterm Infant Gut Microbiota and Metabolome: An Observational Study. *Cell Rep Med*. **1,** (2020)
9. Aguilar-Lopez M. et al. Metagenomic profile of the fecal microbiome of preterm infants consuming mother's own milk with bovine milk-based fortifier or infant formula: a cross-sectional study. *Am J Clin Nutr*. **116**, 435-445 (2022).
10. Jia, Q. et al. Dynamic changes of the gut microbiota in preterm infants with different gestational age. *Front Microbiol* 13 (2022).

11. Korpela, K., et al. Intestinal microbiota development and gestational age in preterm neonates. *Sci rep*. **8**, 1-9 (2018).

12. Datta, M. S., et al. Rapid methicillin resistance diversification in Staphylococcus epidermidis colonizing human neonates. *Nat Commun*. **12**, 1-10 (2021).

13. Dobbler, P. T., et al. Low microbial diversity and abnormal microbial succession is associated with necrotizing enterocolitis in preterm infants. *Front microbiol*. **8**, 2243 (2017).
